# Co-development of a consensus-based pathway to improve access and outcomes for women seeking flat symmetry as an alternative to breast reconstruction after mastectomy for breast cancer: Protocol for the UK FLAME study

**DOI:** 10.1136/bmjopen-2026-120430

**Published:** 2026-07-23

**Authors:** Eleanor Ferris, Sam Brunsden, Katherine Cowan, Katherine Fairhurst, Sue Hartup, Nicola King, Stuart McIntosh, Nicola Mills, Joanna Skillman, Samuel G Smith, Philippa Tollow, Shelley Potter

**Affiliations:** 1 Bristol Surgical and Perioperative Care Complex Intervention Collaboration, Clinical Sciences, Bristol Medical School, Bristol, UK; 2 Flat Friends UK, London, UK; 3 Katherine Cowan Consulting, St Leonards-on-Sea, East Sussex, UK; 4 Bristol Breast Care Centre, Bristol NHS Foundation Trust, Bristol, UK; 5 St James’s University Hospital, Leeds Teaching Hospitals NHS Trust, Leeds, UK; 6 The Johnston Cancer Research Centre, Queen’s University Belfast School of Medicine Dentistry and Biomedical Sciences, Belfast, Northern Ireland, UK; 7 Population Health Sciences, Bristol Medical School, Bristol, UK; 8 Department of Plastic Surgery, University Hospitals Coventry and Warwickshire NHS Trust, Coventry, UK; 9 Leeds Institute of Health Sciences, University of Leeds, Leeds, UK; 10 University of the West of England, Bristol, UK

**Keywords:** Breast tumours, Breast surgery, Health Services Accessibility

## Abstract

**Abstract:**

**Introduction:**

Up to 40% of women with breast cancer will require a mastectomy, and breast reconstruction is routinely offered to restore symmetry. While not all women want, or are suitable for, breast reconstruction, many want to be symmetrical following breast cancer surgery. For these women, contralateral mastectomy for ‘flat symmetry’ is a good alternative, but in the UK, access to contralateral ‘symmetrising’ mastectomy (CSM) is highly variable. Many women seeking this option describe feeling frustrated, unsupported and having to ‘fight’ to access care, while clinicians express concerns about decisional regret. The FLAME (FLat symmetry After Mastectomy for brEast cancer) study aims to work with key stakeholders to co-develop a consensus-based pathway to improve access and outcomes for women seeking CSM as an alternative to breast reconstruction after a unilateral mastectomy for breast cancer.

**Methods and analysis:**

Pathway development will be underpinned by the Medical Research Council (MRC) framework for the development of complex interventions, informed by the Behaviour Change Wheel and Capability, Opportunity, Motivation—Behaviour framework, complemented by Intervention Mapping. There will be six key stages: (1) creation of a logic model of the issues that need to be addressed with the CSM pathway using a systematic literature review, national practice surveys and qualitative interviews with key stakeholders; (2) definition of the logic model of change objectives (performance objectives, change determinants and change objectives); (3) pathway design informed by appropriate behaviour change techniques; (4) co-development of the pathway in a co-design workshop involving key stakeholders; (5) co-development of an implementation plan and (6) future evaluation.

**Ethics and dissemination:**

Ethical approval has been obtained from the University of Bristol Research Ethics Review Committee (ref: 27961). Written and verbal informed consent will be obtained from all participants before the interview study and again from individuals participating in the workshop. Findings will be presented at national/international meetings and published in peer-reviewed journals. Dissemination materials will be co-produced with key stakeholders and shared widely with professional associations and patient support organisations to promote uptake and implementation.

**STRENGTHS AND LIMITATIONS OF THIS STUDY**

- This study aims to develop a consensus-based pathway for women seeking contralateral ‘symmetrising’ mastectomy (CSM) as an alternative to breast reconstruction after a unilateral mastectomy for breast cancer.
- Intervention development will be informed by empirical data from our systematic review, national practice survey and qualitative interviews representing diverse perspectives.
- The pathway will be co-developed by key stakeholders and underpinned by the Medical Research Council’s framework for complex intervention development, but further work will be needed to explore the feasibility and acceptability of the pathway before implementation.
- Recruiting participants through social media and patient advocacy groups risks including individuals with strong views, but involvement of an independent facilitator to oversee the process and careful workshop participant selection should mitigate against this.
- The empirical data used to inform intervention development will only reflect views from the UK, and further work will be needed to establish whether the CSM pathway has utility in other healthcare settings.

## Introduction

 Despite improvements in treatment, approximately 40%^1^ of the 56 000 women diagnosed with breast cancer every year in the UK^2^ will undergo a mastectomy. It is widely recognised that mastectomy can impact body image and quality of life^3^ and the National Institute for Health and Care Excellence (NICE) recommends that all women should routinely be offered breast reconstruction (BR) to restore symmetry.^4^ While BR may be an excellent option for some women, not all are suitable. In addition, many women elect not to have BR^5^ as it can be complex, risk of additional postoperative complications,^6^ involve further surgery^7^ and may prolong recovery.

While not all women want or are suitable for BR, most desire symmetry following breast cancer surgery.^8^ For some, this may be achieved using an external prosthesis, but these can be heavy and uncomfortable,^9^ only achieve symmetry when dressed and may impact women’s confidence to pursue physical activities.^10^ Postmastectomy, some women feel self-conscious or ‘lopsided’^11^ and a lack of symmetry and dissatisfaction with body image can significantly impact quality of life.^12^ Contralateral ‘symmetrising’ mastectomy (CSM), defined as mastectomy performed to achieve symmetry rather than for oncological or risk-reducing indications, offers women the possibility of achieving ‘flat symmetry’ as an alternative to BR. CSM can be performed either at the time of the index mastectomy or as a delayed procedure, and with the support of patient advocacy groups such as ‘Flat Friends’, more women are now seeking this option.

Despite this, our recent national practice survey^13^ highlights considerable variation in how and when CSM is offered, if at all. Women requesting surgery often face resistance from clinicians who are unwilling or reluctant to offer the procedure^14 15^ and few routinely discuss this option with patients.^13 16^ The professional associations do not support offering contralateral ‘risk-reducing’ mastectomy (CRRM) to women with unilateral breast cancer unless they have a high familial or genetic risk^17^ due to a lack of oncological benefits,^18 19^ but this justification is not applicable to women seeking surgery for flat symmetry.^20^ The current failure to differentiate the management of women seeking CSM from that of those requesting contralateral ‘prophylactic’ mastectomy/CRRM is likely to be a major barrier to equitable service provision that needs to be addressed.

Any proposed management pathway, however, should reflect the needs of both patients and healthcare professionals. The recently revised 2021 Oncoplastic Guidelines^21^ suggest that women seeking CSM should be discussed at an oncoplastic multidisciplinary meeting and be seen by psychological services. Although this approach may support clinicians concerned about decisional regret,^22^ qualitative research suggests that a requirement for psychological input can make women feel stigmatised and left ‘questioning their own sanity’.^15^ Our recent systematic review does not provide any evidence to suggest that women regret their decision but suggests that surgeons’ denial of flat symmetry as an option negatively affects their experiences and outcomes.^23^

There is therefore an urgent need to develop a standardised, nationally agreed pathway for women seeking flat symmetry to address the current unacceptable inequity in care and to develop strategies to support professionals to routinely offer CSM, which was recently identified as a top UK research priority.^24^ Codevelopment involving patients and healthcare professionals is vital to ensure that the proposed pathway is both acceptable to patients and supports clinicians in equitably offering CSM as an alternative to BR. The Intervention Mapping framework is an established methodology for developing health programmes^25^ and highlights the importance of codesign, and the use of empirical data and stakeholder input to guide intervention development.^26 27^

### Aim

The FLAME study aims to use the Intervention Mapping framework to codevelop a consensus-based pathway to improve access and outcomes for women seeking CSM as an alternative to BR after a unilateral mastectomy for breast cancer.

## Methods and analysis

### Patient and public involvement

Patients and women with lived experience are integral to this project, which addresses a top research priority for information and support identified with over 200 patients in a recent priority setting exercise.^24^

Patient advocates have been involved with the project from inception and have contributed to all aspects of study design. They will be equal members of the study steering group, which will also include representatives of other key stakeholder groups (breast and plastic surgeons, specialist nurses, psychologists, methodologists and members of support/charitable organisations). This group will be convened at the start of the project and will play a key role in all stages of pathway development. The FLAME steering group and pathway co-design workshop will be chaired by an independent facilitator (KC) with extensive experience of co-development work. This approach will ensure that all views are heard in a fair and balanced way and that no one stakeholder group or perspective is allowed to dominate the process or outcomes.

### Overview

CSM pathway development will be underpinned by the Medical Research Council (MRC) framework for the development of complex interventions,^28^ informed by the Behaviour Change Wheel^29^ and COM-B (Capability, Opportunity, Motivation—Behaviour) framework,^29^ complemented by Intervention Mapping.^26 27^ The COM-B model and Theoretical Domains Framework^30^ will be used to analyse and understand behaviour change and explain the active components and mechanisms required to change behaviour as part of pathway development.

The Intervention Mapping framework will consist of 6 steps as follows:

1. Create a logic model of the issues that need to be addressed by the CSM pathway.
2. Define performance objectives, change determinants and change objectives (logic model of change objectives).
3. Design the pathway by selecting a suitable behaviour change technique (BCT).
4. Develop the pathway components.
5. Develop an implementation plan.
6. Develop an evaluation plan.

### Step 1— Development of a logic model

Findings from our systematic review,^23^ national practice survey^13^ and qualitative interviews will be used to conduct a needs assessment to identify the key challenges for patients seeking CSM and professionals offering CSM that the pathway will need to address together with any proposed solutions, to inform steps 2 and 3.

#### Qualitative interview study

Semi-structured qualitative interviews with key stakeholders will be used to explore and gain an in-depth understanding of the issues around offering CSM and views on the provision of care. Stakeholders will include individuals with lived breast cancer experience, breast/plastic surgeons, specialist nurses, clinical psychologists and members of support organisations (eg, Breast Cancer Now, Flat Friends UK).

##### Eligibility criteria

Women with lived experience defined as having a unilateral mastectomy for breast cancer and who have undergone, are awaiting, have requested or are considering CSM and have completed their initial breast cancer treatment with surgery±chemotherapy ± radiotherapy (in any order) will be eligible to participate in the study. Women receiving long-term adjuvant treatment with CDK4/6 inhibitors (eg, Ribociclib) or endocrine therapy (eg, tamoxifen or aromatase inhibitors) will be eligible to participate, but those with metastatic disease undergoing palliative treatment will be excluded. Women seeking contralateral mastectomy for risk reduction (eg, those with a pathological genetic mutation or at high familial genetic risk) and those seeking reconstructive surgery at the time of their mastectomy will be excluded.

Any healthcare professional involved in the management of women seeking CSM will be eligible to take part. This will include, but not be limited to, breast and plastic surgeons, specialist nurses and clinical psychologists.

Members of any organisation providing support or information for patients with breast cancer (eg, Breast Cancer Now, Flat Friends UK) will also be eligible to take part.

##### Sampling and recruitment

Potential participants will be invited to take part in the interview study via social media, professional associations (eg, Association of Breast Surgery), support/charitable organisations (eg, Flat Friends, Breast Cancer Now) and personal/professional networks. Potential participants will be invited to complete a brief ‘Expression of Interest’ form on REDCap,^31^ including email address, participant group and geographical region of the UK to inform purposive sampling.

For women with lived experience, sampling will ensure maximum variation with regard to age, ethnicity, geographical location and whether they have undergone CSM or are awaiting/wishing to have CSM. For healthcare professionals and members of charities/support organisations, sampling will be informed by our national practice survey^13^ and purposively target units with/without formal pathways for managing women seeking CSM. Maximum variation will also be sought regarding profession, gender, experience and geographical location. Snowball sampling will be used to identify professionals with interest/expertise in this area, and theoretical sampling will be used as the study progresses to target individuals whose views/experiences may provide additional or differing insights to enhance or disprove developing themes.

Purposively selected individuals will be contacted by e-mail and invited to read a participant information sheet and complete a consent form prior to participating in an interview.

##### Data collection

Semi-structured interviews will be conducted by telephone or video call at a time convenient to the study participant. Interviews will be conducted in batches of 5–10 per stakeholder group to enable iterative sampling, data collection and analysis.

The interviews will be conducted by a medically qualified researcher (EF) trained in qualitative methods using a topic guide developed by the study steering group. The topic guide will consider relevant literature, clinical experience and explore attitudes to CSM, barriers and facilitators to offering CSM and support needed for women considering/seeking CSM (online supplemental appendix 1). The topic guide will be iteratively refined as the interviews progress to allow exploration of emerging themes. In professional interviews, it will be emphasised that the discussions relate to contralateral mastectomy performed for symmetry in women at population risk and not risk-reducing surgery in any patient group.

Basic demographic information will be collected during the interviews to ensure the study includes a diverse representation of views. For women with lived experience, this will include age, region of the UK, ethnicity, year of cancer diagnosis and brief information about breast cancer treatments received to allow the pathway of care to be explored. For healthcare professionals and members of charities/support organisations, this will include profession/background, experience and region of the UK they are working in.

It is anticipated that interviews will last between 30 and 60 min. Participants will be assured of confidentiality before, during and after the interview. Field notes will be made during the interview, taking into account non-verbal cues and emotional responses for consideration during analysis.

It is possible some participants may find discussing their experiences of seeking, discussing or offering CSM upsetting. Distress protocols will be in place and include an option to terminate the interview.

All interviews will be digitally recorded using an encrypted recording device and transcribed in full using a University of Bristol-approved transcription service. The accuracy of the transcripts will be checked against the original recording. All transcripts will be anonymised to protect participant confidentiality.

##### Data analysis and sample size

Interview transcripts will be analysed using reflexive thematic analysis^32^ in an iterative process commencing soon after data collection. After familiarisation, transcripts will be systematically assigned codes before generating initial themes, which will inform refinements to the interview topic guides. A proportion of transcripts from different stakeholder groups will be independently coded by an experienced researcher with appropriate clinical and methodological expertise (SP). This will promote methodological rigour and ensure the developing codes are grounded in the data. Developing themes and supporting data will be presented to, and discussed with, the study steering group, which includes patient advocates, at regular intervals during the interview study. This will check interpretation, allow iterative refinement of developing themes, facilitate resolution of disagreements and support reflexive engagement with the data. NVivo15 (Lumivero, Denver, USA) will be used to support data analysis.

There is no predefined sample size for qualitative studies. Data collection will be guided by information power, continuing until the dataset is deemed sufficiently rich enough to understand the issues around accessing and offering CSM. Based on the complexity of the topic and diversity of key stakeholder views, we anticipate that up to 50 interviews will be required.

### Step 2— Logic model of change objectives

A logic model of change, informed by our empirical work (systematic review,^23^ national practice survey^13^ and qualitative interviews), will be developed to describe behavioural determinants that the CSM pathway aims to change. Matrices of change objectives will be developed to inform the model, which will illustrate proposed relationships between theory- and evidence-based change methods, the determinants they are expected to influence, and the behavioural outcomes that will support clinicians to routinely discuss CSM with patients. This will underpin the conceptualisation and design of the pathway. BCTs will be linked to the change objectives and collated into an initial design document with input from steering group members. Data from qualitative interviews and surveys will be mapped onto the framework to allow a detailed understanding of possible mechanisms for behaviour change. Preferences and practical considerations will be combined with theory to optimise intervention acceptability and suitability for implementation into practice.

### Step 3— Intervention design

The logic model of change will underpin the conceptualisation and design of the CSM pathway.^28^ The steering group will develop an initial framework for the CSM pathway, identifying key themes/components, scope and sequence required. Empirical data,^13 23^ including those from qualitative interviews, will be mapped onto the framework to encourage a detailed understanding of possible mechanisms for behaviour change.

### Step 4—Intervention production

The BCTs and practical applications will be translated into creative, operational materials that promote the key messages of the intervention. An initial version of the CSM pathway will be cocreated with the steering group and iteratively refined in a 1-day face-to-face co-design workshop with a wider multidisciplinary group of stakeholders.

#### Co-design workshop

##### Sampling and recruitment

All interview participants will be invited to express an interest in taking part in the workshop. Between 20 and 25 interested interview participants will be purposively selected to take part based on stakeholder group, geographical region, age, gender, ethnicity and for women with lived experience, whether they have undergone/are awaiting CSM to ensure the broadest representation of views at the workshop. Formal written consent will be obtained from all participants prior to the workshop.

##### Structure and content

Participants will be sent a ‘meeting pack’ in the weeks before the workshop via e-mail. This will include the meeting agenda, a summary of the empirical research findings, a list of potential components of a future CSM pathway and a proposed initial CSM pathway for discussion.

Travel expenses will be provided, and reasonable adjustments will be made to allow inclusive participation. No video or audio recordings of the meeting will be made. Participants will be informed that all discussions of personal experiences must be kept confidential and will agree to this by signing the consent form. Distress protocols for participants in all stakeholder groups will include an option to leave the workshop and ensure further support is in place.

Acceptability, usability and content of the proposed CSM pathway will be explored. The workshop will be chaired by an independent facilitator with experience in codevelopment who will lead the discussions and voting. The final workshop structure will depend on the proposed CSM pathway but will likely involve a brief presentation outlining the study, findings of the empirical work, the proposed content/structure of the CSM pathway and aims of the meeting. It is anticipated that there will be iterative rounds of small group work, discussion and voting until the final content and structure of the CSM pathway are agreed together with preliminary plans for evaluation. Following the workshop, a final design document will be created to provide a complete overview of the final content of the CSM pathway, linking it to corresponding change objectives and BCTs.

### Steps 5 and 6 — CSM pathway implementation and evaluation

A proposal for a mixed-methods study will be developed to explore the feasibility and acceptability of the CSM pathway, allow further refinement and provide preliminary evidence of effectiveness before implementation. The study design will be informed by the co-design work and pathway structure. Feasibility outcomes are likely to include uptake of pathway resources and measures of patient and clinician experience.

### Study timelines

Ethical approvals were obtained on 19 December 2025, and recruitment to the qualitative study commenced in January 2026. Recruitment and analysis are ongoing. The co-design workshop is planned for September 2026, following which a proposal for the mixed-methods will be developed in early 2027.

## Ethics and dissemination

Ethical approval for the project has been obtained from the University of Bristol WS1 Research Ethics Review Committee (ref: 27961). Written and verbal informed consent will be obtained from all participants before the interview study and again from individuals participating in the workshop.

The findings will be presented at national and international meetings and published in peer-reviewed journals. Dissemination materials will be co-created with our steering group and patient advocates and shared widely via patient support groups, professional networks and social media. We work with the UK professional associations to optimise uptake and implementation of the pathway into clinical practice to support clinicians to equitably offer CSM as an alternative to BR and improve access and outcomes for women seeking this option in the future.

## Supplementary material

10.1136/bmjopen-2026-120430 online supplemental file 1
