# Supplementary material for: Co-development of a consensus-based pathway to improve access and outcomes for women seeking flat symmetry as an alternative to breast reconstruction after mastectomy for breast cancer: Protocol for the UK FLAME study
Source: BMJ Open. 2026 Jul 23;16(7):e120430. doi: 10.1136/bmjopen-2026-120430 (PMC13404634; doi:10.1136/bmjopen-2026-120430)
Supplement: online supplemental file 1 [file bmjopen-16-7-s001.docx]

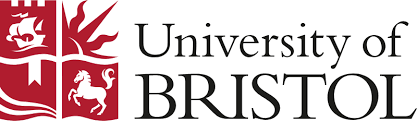
 **
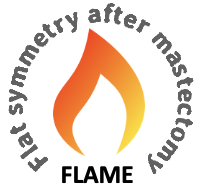
**

**Interview Topic Guide**

Introduction

Greet and confirm who are, iterate that can stop at any point, but anticipate will take 30-60 minutes

Reconfirm consent verbally to audio record interview

Basic demographics

**Patients:** Age | Ethnicity | Area of country treated

**Professionals:** Profession | Gender | Clinical experience | Location working in

For Patients

1. **Tell me about your breast cancer diagnosis. What was your experience?**

When was your breast cancer diagnosed?

Did you have the option of anything other than a mastectomy to remove your breast cancer?

Why did you choose a mastectomy (if offered the choice of mastectomy vs breast conservation)?

What additional treatments did you need? (chemotherapy, surgery)

Were you offered immediate breast reconstruction? Delayed reconstruction? Symmetrising mastectomy mentioned?

How did you feel about this? Explore reasons for not having immediate reconstruction

What influenced your decision-making?

1. **When did you know you wanted a balancing mastectomy for symmetry as opposed to a reconstruction?**

Tell me about your experience? Offered surgery by team vs raising it with clinical team

What happened?

Were there any difficulties/challenges you experienced?

What do you think could be done differently?

What support did you get making the decision? Anything additional you felt you needed?

What process did you have to go through to have surgery? Psychology/plastics etc

How could the process be improved?

In an ideal world, what should happen if a patient asked for a balancing mastectomy for surgery?

Do you think there should be any restrictions on this??

What guidance should we be giving to clinicians?

1. **Do you have anything else to add about your thoughts or experiences or surgical pathway?**

Any questions for me?

For Professionals

**1. What is your experience of contralateral symmetrising mastectomy (CSM)?**

Patient requesting? Performing? Outcomes/follow up? Concerns?

Do you routinely offer it?

How do you feel about offering it?

Concerns??

**2. Can you tell me about the patient pathway for CSM where you work?**

Do patients routinely get offered CSM or is it only discussed if the patient requests?

Is CSM routinely funded, or do you have to apply for exception funding?

Is there are formalised pathway or set of steps patients have to go through to receive CSM? (Psychology, oncoplastic MDT, cooling off period etc)

What are your thoughts about offering and performing CSM?

**3. What do you think a national CSM patient pathway should look like?**

e.g. ? Psychology input, oncoplastic MDT, consider reconstruction also, cooling off period (fixed/flexible/patient dependent)

Do you think patients should be offered CSM at the same time as their index mastectomy?

What support do you think patients need to make the decision for CSM?

Will a national pathway help your practice?

**4.** **Do you have anything else to add about your thoughts or experiences or a CSM surgical pathway?**

Any questions for me?

Close

Thank them for their time taking part in an interview

Study is progressing and we hope to hold a consensus meeting involving representatives from both patient and professional groups – are they interested in being invited to this?
