# Supplementary material for: Co-development of a consensus-based pathway to improve access and outcomes for women seeking flat symmetry as an alternative to breast reconstruction after mastectomy for breast cancer: Protocol for the UK FLAME study
Source: BMJ Open. 2026 Jul 23;16(7):e120430. doi: 10.1136/bmjopen-2026-120430 (PMC13404634; doi:10.1136/bmjopen-2026-120430)
Supplement: Supplementary file 2 [file bmjopen-2026-120430.reviewer_comments.pdf]

PEER REVIEW HISTORY

BMJ Open publishes all reviews undertaken for accepted manuscripts. Reviewers are asked to complete a checklist review form and are provided with free text boxes to elaborate on their assessment. These free text comments are reproduced below.

ARTICLE DETAILS

Title (Provisional)

Co-development of a consensus-based pathway to improve access and outcomes for women seeking flat symmetry as an alternative to breast reconstruction after mastectomy for breast cancer: protocol for the UK FLAME study

Authors

Ferris, Eleanor; Brunsden, Sam; Cowan, Katherine; Fairhurst, Katherine; Hartup, Sue; King, Nicola; McIntosh, Stuart; Mills, Nicola; Skillman, Joanna; Smith, Samuel; Tollow, Philippa; Potter, Shelley

---

VERSION 1 - REVIEW

**Reviewer**	**1**
**Name**	**Cavalcante, Francisco Pimentel**
**Affiliation**	**Hospital Geral de Fortaleza**
**Date**	**22-May-2026**
**COI**	**None**

This is a relevant and well-constructed protocol addressing an important area in breast cancer survivorship .

The manuscript is generally well written and methodologically sound. The topic is highly relevant given the increasing number of women seeking “flat symmetry” as an alternative to breast reconstruction and the reported inequities in access to contralateral symmetrising mastectomy (CSM).

I have some comments that may help strengthen the manuscript.

1. Clarification of terminology: CSM vs CPM/CRRM

Feels like the terminology occasionally remains overlapping throughout the manuscript.

Maybe providing a formal operational definition of CSM early in the Introduction….distinguishing aesthetic/symmetry-driven surgery from oncologic risk-reducing procedures;

how clinicians participating in the study will be guided to differentiate these entities during interviews and workshop discussions ?

2. More detail needed regarding qualitative :

• How will reflexivity be addressed?

• Will coding be performed independently by more than one researcher?

• How will disagreements in coding/theme generation be managed?

• Will patient advocates participate directly in coding or interpretation?

• How will the research team account for potentially strong pre-existing views among advocacy group members?

3. selection bias

Recruitment through advocacy groups and social media may preferentially capture participants with strong views or negative experiences regarding access to CSM.

The manuscript should acknowledge this more explicitly as a potential limitation

4. Lack of discussion regarding surgical risks and resource implications

5. Clarification regarding future evaluation outcomes

The manuscript states that a future mixed-methods study will evaluate feasibility, acceptability and preliminary effectiveness, but no examples of potential outcome measures are provided.

Minor comments

1. Clarify timeline/status of the study

As this is a protocol paper, the manuscript should explicitly state:

• when recruitment is expected to begin/end;

• whether interviews are already ongoing;

• anticipated timeline for pathway development.

BMJ Open specifically requests clarification regarding study status in protocol submissions.

2. Consider discussing international applicability

The authors appropriately acknowledge that empirical data are UK-based. However, a brief discussion regarding the potential transferability of findings to other healthcare systems would be useful.

3. Typographical/editorial suggestions

• Some abbreviations are introduced multiple times (e.g., CSM).

• Minor formatting inconsistencies are present around references and spacing.

• The phrase "questioning their own sanity" may benefit from contextualisation as a direct patient perspective/quotation.

VERSION 1 - AUTHOR RESPONSE

<u>**Reviewer comments**</u>

This is a relevant and well-constructed protocol addressing an important area in breast cancer survivorship. The manuscript is generally well written and methodologically sound. The topic is highly relevant given the increasing number of women seeking "flat symmetry" as an alternative to breast reconstruction and the reported inequities in access to contralateral symmetrising mastectomy (CSM). I have some comments that may help strengthen the manuscript.

We thank the Reviewer for their kind comments. We have addressed the issues raised below.

1. Clarification of terminology: CSM vs CPM/CRRM. Feels like the terminology occasionally remains overlapping throughout the manuscript. Maybe providing a formal operational definition of CSM early in the Introduction….distinguishing aesthetic/symmetry-driven surgery from oncologic risk-reducing procedures; how clinicians participating in the study will be guided to differentiate these entities during interviews and workshop discussions?

Thank you for asking for additional clarification on this point.

We have revised the introduction (page 4 para 2) for clarity. This now reads:

'Contralateral 'symmetrising' mastectomy (CSM), <u>***defined as mastectomy performed to achieve symmetry rather than for oncological or risk-reducing indications***</u>, offers women the possibility of achieving 'flat symmetry' as an alternative to breast reconstruction.'

All study documentation describes CSM as contralateral mastectomy performed for symmetry in women at population breast cancer risk. It is clear throughout that CSM is for symmetry rather than for risk reduction or other oncological indications. We have added an additional sentence to the 'Data collection' section of the 'Methods' (page 8, para 2) to state:

<u>***'In professional interviews, it will emphasised that the discussions relate to contralateral mastectomy performed for symmetry in women at population risk and not risk reducing surgery in any patient group.'***</u>

2. More detail needed regarding qualitative:
• How will reflexivity be addressed?

Thank you for requesting more information regarding reflexivity and how this will be addressed. We have provided clear information regarding the background of the researcher undertaking the interviews (page 9) and about how the topic guide was developed (page 9). We have highlighted that field notes will be made during the interviews taking into account the 'non-verbal cues and emotional responses' for consideration in the analysis (page 10).

A proportion of transcripts will be dual coded by an experienced researcher with clinical and methodological expertise to promote methodological rigor and ensure that developing codes are grounded in the data. Developing themes and supporting data will be presented to the steering group at regular meetings and the themes developed and iteratively refined based on feedback received. This is stated in the 'Data analysis and sample size' section (page 10).

This now reads:

'Interview transcripts will be analysed using reflexive thematic analysis[32] in an iterative process commencing soon after data collection. After familiarisation, transcripts will be systematically assigned codes, before generating initial themes which will inform refinements to the interview topic guides. ***A proportion of transcripts from different stakeholder groups will be independently coded by an experienced researcher with appropriate clinical and methodological expertise (SP). This will promote methodological rigor and ensure the developing codes are grounded in the data. Developing themes and supporting data will be presented to, and discussed with, the study steering group which includes patient advocates, at regular intervals during the interview study. This will check interpretation, allow iterative refinement of developing themes, facilitate resolution of disagreements and support reflexive engagement with the data***. NVivo15 (Lumivero, Denver USA) will be used to support data analysis.'

• Will coding be performed independently by more than one researcher?

As stated above, a proportion of the transcripts will be dual coded by an experienced researcher with clinical and methodological expertise. Developing themes will also be discussed with the wider steering group at regular meetings.

• How will disagreements in coding/theme generation be managed?

Developing themes and supporting data will be presented to and discussed with the study steering group at allow disagreements in interpretation to be resolved (see above)

• Will patient advocates participate directly in coding or interpretation?

Patient advocates are equal members of the study steering group. As stated above, developing themes and supporting data will be presented to the steering group at regular intervals throughout the qualitative study. They will contribute to interpretation and iterative refinement of developing themes, ensuring the patient perspective remains at the heart of this project. We have added that the steering group includes patient advocates to this is clear (see above).

• How will the research team account for potentially strong pre-existing views among advocacy group members?

We appreciate that this is a contentious and emotive topic for both patients and clinicians, so have recruited an independent facilitator (KC) to chair the steering group and co-design workshops and oversee the process. She will ensure that everyone's views are taken into account without allowing any individual or stakeholder group to dominate the process.

We had added some additional information regarding this in “Patient and Public Involvement’ section. Page 6, para 2 now reads:

‘The FLAME steering group and pathway co-design workshop will be chaired by an independent facilitator (KC) with extensive experience of co-development work. This approach will ensure that all views are heard in a fair and balanced way and that no one stakeholder group or perspective is allowed to dominate the process or outcomes.’

3. Selection bias. Recruitment through advocacy groups and social media may preferentially capture participants with strong views or negative experiences regarding access to CSM. The manuscript should acknowledge this more explicitly as a potential limitation

Thank you for highlighting this issue. We have included this as a bullet point in the ‘Strengths and Limitations’ section of the manuscript but also suggested that involvement of an independent facilitator to oversee the process and careful workshop participant sampling should mitigate against this.

4. Lack of discussion regarding surgical risks and resource implications

We agree that surgical risks and resource implications are likely to be key issues raised by healthcare professionals, but we do not wish to pre-empt the study by suggesting what may need consideration as part of the pathway. We have already noted within the “Logic Model of Change Objectives” section that *“Preferences and practical considerations will be combined with theory to optimise intervention acceptability and suitability for implementation into practice.”* (page 11). We hope that this is sufficient.

5. Clarification regarding future evaluation outcomes
The manuscript states that a future mixed-methods study will evaluate feasibility, acceptability and preliminary effectiveness, but no examples of potential outcome measures are provided.

The details of a future mixed-methods study will be informed by the co-development work and pathway structure and content. It is not possible to provide any definitive outcome measures at this stage.

We have added a sentence to the ‘CSM pathway implementation and evaluation’ section for further clarification.

Step 5 & 6 (page 13) now read:

‘The study design will be informed by the co-design work and pathway structure. Feasibility outcomes are likely to include uptake of pathway resources and measures of patient and clinician experience.’

Minor comments

1. Clarify timeline/status of the study

As this is a protocol paper, the manuscript should explicitly state:
• when recruitment is expected to begin/end;
• whether interviews are already ongoing;
• anticipated timeline for pathway development.
BMJ Open specifically requests clarification regarding study status in protocol submissions.

We have added a 'Timelines' section at the end of the methods (page 13) which reads:

Timelines
Ethical approvals were obtained 19th December 2025 and recruitment to the qualitative study commenced in January 2026. Recruitment and analysis are ongoing. The co-design workshop is planned for September 2026 following which a proposal for the mixed methods will be developed early 2027.

2. Consider discussing international applicability
The authors appropriately acknowledge that empirical data are UK-based. However, a brief discussion regarding the potential transferability of findings to other healthcare systems would be useful.

We have added additional information to the 'Strengths and Limitations' section of the manuscript to reflect this, however, this is a protocol paper. There is not a formal discussion section within the manuscript, so it is not possible to discuss potential transferability to other healthcare settings. Furthermore, as it is not yet clear what the structure and content of the pathway will be, it is not possible to draw any conclusions regarding transferability beyond those already stated.

3. Typographical/editorial suggestions
• Some abbreviations are introduced multiple times (e.g., CSM).
• Minor formatting inconsistencies are present around references and spacing.
• The phrase "questioning their own sanity" may benefit from contextualisation as a direct patient perspective/quotation.

We have corrected these typographical errors and formatting inconsistencies. We have contextualised the phrase "questioning their own sanity" by amending the sentence to *"[…]* ***qualitative research suggests that a requirement for*** *psychological input can make women feel stigmatised and left 'questioning their own sanity'."* (page 5).

We hope that the above responses and revisions meet with your satisfaction and that you will now reconsider the manuscript for publication in the BMJ Open.

VERSION 2 - REVIEW

**Reviewer**	**1**
**Name**	**Cavalcante, Francisco Pimentel**
**Affiliation**	**Hospital Geral de Fortaleza**

Date **13-Jul-2026**

COI

The authors have adequately addressed all reviewer comments. The manuscript has been substantially strengthened, particularly regarding methodological transparency.
